# Prevalence and Correlation between TMD Based on RDC/TMD Diagnoses, Oral Parafunctions and Psychoemotional Stress in Polish University Students

**DOI:** 10.1155/2014/472346

**Published:** 2014-07-09

**Authors:** Mieszko Wieckiewicz, Natalia Grychowska, Kamil Wojciechowski, Anna Pelc, Michal Augustyniak, Aleksandra Sleboda, Marek Zietek

**Affiliations:** ^1^Division of Dental Materials, Faculty of Dentistry, Wroclaw Medical University, 26 Krakowska Street, 50425 Wroclaw, Poland; ^2^Faculty of Dentistry, Wroclaw Medical University, 26 Krakowska Street, 50425 Wroclaw, Poland; ^3^Department of Periodontology, Faculty of Dentistry, Wroclaw Medical University, 26 Krakowska Street, 50425 Wroclaw, Poland

## Abstract

The aim of the study was to assess the prevalence of temporomandibular disorders (TMD) and oral parafunctions, as well as their correlation with psychoemotional factors in Polish university students. The research was conducted in a group of 456 students (*N* = 456). The examination form comprised of two parts: survey and clinical examination. The research diagnostic criteria for temporomandibular disorders (RDC/TMD) was used in order to assess TMD. Symptoms of TMD were observed in 246 (54%) students after clinical examination. The largest group involved students with disc displacement (women: 132, 29%; men: 70, 15%). Women (164; 36%) suffered more frequently than men (82; 18%) from problems related to the stomatognathic system (*P* < 0.05), described themselves as easily excitable and emotionally burdened, and reported symptoms as tightness of the facial and neck muscles (*P* < 0.05). In 289 (64%) students intraoral symptoms concerning occlusal parafunctions were observed. In 404 (89%) examined students, nonocclusal parafunctions were recorded. A significant correlation between TMD and psychoemotional problems could be detected. TMD symptoms more often concern women. Emotional burden and excitability are factors predisposing muscular disorders.

## 1. Introduction

The temporomandibular disorders (TMD) and oral parafunctions seem to be a frequent problem in modern societies [1–6]. The etiopathology of the temporomandibular joints is related to muscles, teeth arches, and periodontium. Their main causes involve both pathophysiological and psychosocial factors [7, 8]. In the literature, a significant impact of the psychoemotional factor is reported, comparable to the impact of other factors concerning physical health such as systemic diseases, malocclusions, loss of teeth, traumas, and microtraumas [9–12]. Stress, fatigue, anxiety, depression, sleep disorders, and a fast pace of life affect negatively the human psyche [13]. In those patients muscular related TMD is observed more often [14]. Moreover different studies report that TMD coexists with other numerous disorders such as SAPHO syndrome (synovitis, acne, pustulosis, hyperostosis, and osteitis syndrome), fibromyalgia, back- or spine ache, chronic fatigue syndrome, spastic colons, sleep disorders, congenital defects, headaches, and arthritis [11, 12, 15, 16]. Many studies report that the symptoms of the masticatory system disorders are more frequent in women than in men [2–5]. This may result from biological differences, including hormonal ones, and also psychosocial factors [17, 18]. Stallman reports that student population lives more under stress than the general population and develops considerably often TMD and oral parafunctions [19]. The possible stress factors include poor learning achievements, financial problems, unergonomic body position while studying, exams, submitting academic papers, and the necessity to become independent [19–21]. Another predisposing factor to the occurrence of the masticatory system disorders in students is the age of this population, since the peak of the development of the symptoms is between 20 and 40 years of age [3].

The aim of this epidemiological study is to assess the prevalence of temporomandibular disorders and oral parafunctions among Polish university students and their correlation with psychoemotional factors.

## 2. Materials and Methods

The study was conducted at the four different Polish universities (Wroclaw Medical University, Wroclaw University of Technology, Wroclaw University of Environmental and Life Sciences, and University of Wroclaw) on a group of 456 students in the years 2012–2014 (N = 456). Inclusion criteria were that the participants should be aged between 19 and 30 years, be Polish university student (both gender were acceptable), and express consent to participate voluntarily in the study. Two hundred sixty four (58%) women and 192 (42%) men were examined. The mean age of participants was 22.01 ± 2.11 years.

The analysis was conducted by the research diagnostic criteria for temporomandibular disorders (RDC/TMD) introduced by Dworkin and LeResche in 1992 [1]. This enables the standardization of the procedures of epidemiological studies, the unification of TMD diagnostic and exploratory criteria, and the comparison of results of other similar studies. The results of the study were based on the RDC/TMD Axis I diagnostic criteria. Mental state of subjects was not assessed according to RDC/TMD Axis II diagnoses.

Before the commencement all researchers have been trained and calibrated in accordance with the adopted norms presented on the official website of the International RDC/TMD Consortium [22]. A simplified version of the examination form was used, which comprised of the survey and the clinical examination, developed on the basis of the RDC/TMD clinical physical examination form and questionnaire [23].

As first part the survey included questions concerning basic demographic data such as gender and age, type of reaction to stress, emotional burden, and occurrence of subjective symptoms of TMD such as fatigue and tightness of the facial muscles, cervical muscle pain, chronic headache, and otologic symptoms. It also included questions concerning the occurrence of nonocclusal parafunctions (habitual gum chewing, lip biting, nail biting and/or skin biting around nails, objects biting, and cheek biting).

During the second part of the clinical examination, patients were given one or more diagnoses according to RDC/TMD Axis I: group I/muscle disorders, group II/disc displacements, and group III/other common joint disorders. The study did not take into account individual unilateral diagnosis of TMD. Over the clinical examination no reliable diagnosis was possible in order to unambiguously group a patient to subgroups IIc, IIIb, or IIIc. Thus, the authors used only groups IIa, IIb, and IIIa. Intraoral symptoms of occlusal parafunctions as teeth attrition, buccal linea alba, gingival recession, crenated tongue, and cracked enamel were systematically documented in an assessment form specially made for the study.

Based on self-report, clinical criteria, and diagnosis, the patients were divided in the following groups.

*Group I: Muscle Disorders*

*Ia Myofascial Pain*

Consider the following:

- pain or ache in the jaw, temples, face, preauricular area, or inside the ear at rest or during function;
- pain in response to palpation of three or more of the following muscle sides (right and left side count as separate sides for each muscle): posterior temporalis, middle temporalis, anterior temporalis, origin of masseter, insertion of masseter, posterior mandibular region, submandibular region, lateral pterygoid area, and tendon of the temporalis;
- at least one of painful muscle must be homolateral to the reported pain.

*Ib Myofascial Pain with Limited Opening*

Consider the following:

- myofascial pain as defined in Ia;
- pain-free unassisted mandibular opening <40 mm interincisal distance;
- maximum assisted opening (passive stretch) of ≥50 mm/greater than pain-free unassisted opening.

*Group II: Disc Displacements*

*IIa Disc Displacement with Reduction*

Consider the following:

- clicking in temporomandibular joint (TMJ) (click on both vertical opening and closing/occuring at a point at least 5 mm greater interincisal distance on opening than closing/eliminated on protrusive opening), reproducible on 2 of 3 consecutive trials;
- clicking in TMJ on both vertical range of motion (either opening or closing), reproducible on 2 of 3 consecutive trials and clicking during lateral excursion or protrusion, reproducible on 2 of 3 consecutive trials.

*IIb Disc Displacement without Reduction with Limited Opening*

Consider the following:

- history of locking or catching that interfered with eating;
- unassisted opening (even painful) ≤35 mm interincisal distance;
- contralateral excursion <7 mm and/or uncorrected deviation to ipsilateral side on opening;
- absence of joint sound or presence of joint sounds not meeting criteria for disc displacement with reduction.

*Group III: Other Common Joint Disorders*

*IIIa Arthralgia*

Consider the following:

- painful joint uni- or bilateral (lateral pole and/or posterior attachment) during palpation;
- one or more painful regions (self-report): pain in the joint, pain in the joint during maximum unassisted opening, pain in the joint during assisted opening, and pain in the joint during lateral excursion;
- coarse crepitus must be absent for simple arthralgia diagnosis.

The intergroup differences were tested using the chi-square test for independence and the test of difference between two proportions as appropriate. Statistical significance level was set at *P* < 0.05. Statistica 10.0 was used in the conducted analysis (StatSoft Inc., Tulsa, Oklahoma, USA).

## 3. Results

### 3.1. Results of the First Part-Survey

From 456 examined students 194 (43%) described themselves as easily excitable, 263 (58%) suffered from emotional burden, 139 (30%) reported fatigue and/or tightness of the facial muscles, 214 (47%) cervical muscle pain and 180 (40%) students regular headaches. Significantly more women than men reported the above symptoms (*P* < 0.05). Otologic symptoms are declared by 87 students (19%) (Table 1). Nonocclusal parafunctions were reported from 404 (89%) examined students. In the majority of cases no significant differences in the prevalence of nonocclusal parafunctions between genders could be detected, excluding lip biting, which is more frequent in women (Table 2).

Students who perceive themselves as easily excitable and/or as having chronic emotional burdens also reported more often fatigue and tightness of facial muscles, cervical muscle pain and headaches compared to other participants (*P* < 0.05). The prevalence of those symptoms were, respectively, 77 (17%), 111 (24%), and 88 (19%) in the group of students defining themselves as excitable, while in the group suffering from chronic emotional burdens 111 (24%), 154 (34%), and 126 (28%).

### 3.2. Results of the Second Part-Clinical Examination

In the second part of clinical examination TMD symptoms were found in 246 (54%) students. More often were detected students with symptoms of disc displacements. Almost one third (147; 32%) of subjects were classified in more than one diagnostic group; no one represented all TMD symptoms (Table 3). Significantly more women (164; 36%) than men (82; 18%) suffered from stomatognathic system problems (*P* < 0.05). The prevalence of TMD symptoms and their correlation to the gender are presented in Table 4.

In group I/muscular disorders a significant higher percentage belonged to students with emotional burden and/or excitability, suffering from fatigue and tightness of facial muscles, cervical pain, and headaches (for all *P* < 0.05; Table 5).

In 289 (64%) students intraoral symptoms concerning occlusal parafunctions were observed (women: 166, 37%; men: 123, 27%). In most cases no differences between men and women were found, with the exception of a higher prevalence of crenated tongue in women (*P* < 0.05). No correlation was recorded between the frequency of the symptoms of occlusal parafunctions and excitability, chronic emotional burdens, fatigue, tightness of facial muscles, cervical muscle pain, and headaches, although a significant correlation between crenated tongue and students with regular headaches was observed (*P* < 0.05).

## 4. Discussion

Temporomandibular disorders have a multifactorial etiopathogenesis. Several authors underline the influence of local factors on their development, while others underline that of systemic factors [24, 25]. The importance of psychological factors, such as increased psychoemotional activity and stress, is also emphasized in literature describing the etiology of TMD and oral parafunctions [26, 27]. In the presented study we also observed a significant role of psychoemotional factors in the TMD development. Easily excitable and emotionally burdened persons suffered significantly more often from TMD and oral parafunctions. It must be mentioned that the RDC/TMD Axis I protocol includes valid diagnostic criteria for differentiating the patients in this study into two groups, with and without TMD. Although this protocol served the scope of the study, generally enables the diagnosis of a limited number and common TMD.

Stress is also an important factor contributing to the TMD development. The examined student population is particularly susceptible to the influence of this factor [28]. Stressors may include a large number of duties, the pressure of getting a good education, an uncertain future, low income, living far away from home, and functioning in an alien environment. Moreover, students also face social, emotional, physical, and family problems [27]. Over half of the students in this study (58%) identified themselves as emotionally burdened. According current literature this percentage could reach 72% [29] or even 90% [30] in student population. It has been proved that being under stress increases the activity of the masticatory muscles, which consequently results in TMD [31].

Further, some studies underline also the gender influence on TMD development [32, 33]. An intercontinental research in 17 countries on 85052 adults supports that 62% of women and 38% of men suffer from TMD [34]. In similar studies in student populations the respective percentage is 65% for women and 35% for men [32, 34]. In the presented study, a greater disproportion between the different genders (70% women; 30% men) was detected, showing that women are more susceptible to TMD than men. This may result from the hormone level fluctuation, biological differences, social position, or higher sensitivity to pain in women [35]. The peak of the development of the symptoms is between 20 and 40, in a reproductive period [17, 36]. It may be caused by a greater number of estrogen and progesterone receptors in intra-articular cartilage in women with TMD. Thorn et al. refers that women report even slight pain during clinical examination, while men only when it is intensified, which could in a way contribute to achieving the above results [37]. This study has also indicated women as a group in which TMD is more frequent in all three diagnostic groups (I–III).

The most frequent disorder of TMJ in the examined population is disc displacement (44%). Similar results were obtained by Karibe et al. [38] in patients aged between 16 and 18 (46.3%), Miyake et al. [39] in patients with mean age 20 years (41.7%) and Poveda-Roda et al. [40] in a group of people with mean age 40 years (51.3%). The study of Manfredini et al. [41] in patients with mean age 32 years did not get a clear result stating a dominance of one of the disorders, while Guarda-Nardini et al. [42] in a group with average age of 41 reported a higher prevalence of pain in TMJ (61.4%). Only Karibe et al. [38] assessed the frequency of type I disorders on the same level as type II in a group of subjects aged between 16 and 18. Stress, which is the main cause of the stomatognathic system disorders, is characteristic for young people up to 20 years old and for patients who are in their 40's, but the reasons of tension in each age group vary [38]. The comparison of data obtained in this and the above referred studies are presented in Table 3.

Not everyone is equally susceptible to the development of oral parafunction. For example, genetic factors affect the development of bruxism, since the significant influence of genetic polymorphism of receptor gene 5HT2 and particularly the presence of allele C rs6313 has been proved [43]. Further, the psychological factor and stress play also a significant role [44]. Each person perceives the world objectively and deals with stress differently depending on its psyche. Neurotic persons often relieve stress by straining the stomatognathic system through teeth clenching, contracting the masticatory muscles, and teeth grinding [14]. Consequently, an interdisciplinary approach from both dental clinicians and psychologists is prerequisite for a successful therapy in patients suffering from stomatognathic system disorders. Any therapeutic effort should exclude the cause, which in case of TMD and oral parafunctions is a failure to deal with stress effectively in everyday life.

## 5. Conclusions

1. The prevalence of TMD in studied Polish student population is 54%.
2. TMD symptoms more often concern women.
3. Emotional burden and excitability are factors that predispose to muscular disorders.
4. The results based on RDC/TMD diagnoses are in accordance with current literature.

## Figures and Tables

**Table 1:** Answer distribution in questionnaire-first part/survey.

Gender	Feature											
	Excitability		Emotional burden		Fatigue and tightness of facial muscles		Cervical muscle pain		Headache		Otologic symptoms	
	Yes	No	Yes	No	Yes	No	Yes	No	Yes	No	Yes	No
Women	124∗ (27%)	140 (31%)	168∗ (37%)	96 (21%)	91∗ (20%)	173 (38%)	152∗ (33%)	112 (25%)	138∗ (30%)	125 (27%)	56 (12%)	208 (46%)
Men	70 (15%)	122 (27%)	95 (21%)	96 (21%)	48 (11%)	144 (32%)	62 (14%)	130 (29%)	42 (9%)	150 (33%)	31 (7%)	161 (35%)

Total	194 (43%)	262 (57%)	263 (58%)	192 (42%)	139 (30%)	31 (7%)	214 (47%)	242 (53%)	180 (40%)	275 (60%)	87 (19%)	369 (81%)

*P < 0.05 comparison between women and men; (%) percentage off all respondents.

**Table 2:** Prevalence of nonocclusal parafunctions in tested population.

Gender	Feature						
	Total	Gum chewing	Lip biting	Nail biting	Skin biting around nails	Objects biting	Cheek biting
Women	238 (52%)	199 (44%)	148 (32%)∗	49 (11%)	105 (23%)	95 (21%)	111 (24%)
Men	166 (36%)	133 (30%)	87 (19%)	39 (9%)	63 (14%)	58 (13%)	71 (16%)

Total	404 (89%)	332 (73%)	235 (52%)	88 (19%)	168 (37%)	153 (34%)	182 (40%)

*P < 0.05 comparison between women and men; (%) percentage off all respondents; respondents declared more than one parafunction.

**Table 3:** Distribution of single and combined RDC/TMD diagnoses in the tested population compared to the results of other studies.

Author	Feature								
	Mean age ± SD	Total *n*	I *n* (%)	II *n* (%)	III *n* (%)	I + II *n* (%)	I + III *n* (%)	II + III *n* (%)	I + II + III *n* (%)
Achieved results	22,01 ± 2,11	456	82 (18,0)	202 (44,3)	81 (17,8)	53 (11,6)	42 (9,2)	52 (11,4)	0
Karibe et al. [38]	17 ± 0,9	167	59 (48,8)	56 (46,3)	6 (4,9)	—	—	—	—
Miyake et al. [39]	20,4 ± 2,1	3557	—	1483 (41,7)	569 (16)	—	—	—	—
Poveda-Roda et al. [40]	40,5 ± 18,7	850	299 (35,2)	436 (51,3)	115 (13,4)	—	—	—	—
Manfredini et al. [41]	32,7 ± 14,5	243	9 (4,5)	24 (12,1)	38 (19,1)	4 (2)	38 (19,1)	43 (21,6)	43 (21,6)
Guarda-Nardini et al. [42]	41,7 ± 17	383	16 (12,6)	27 (21,3)	78 (61,4)	—	—	—	—

*n* number of examined; (%) percentage of examined; “—” not included in the study.

**Table 4:** Prevalence of TMD symtpoms according to the gender.

Gender	Feature						
	GROUP I			GROUP II			GROUP III
	Muscle disorders			Disc displacements			Arthralgia
	I (total)	Ia	Ib	II (total)	IIa	IIb	IIIa
Women	63 (14%)^∗$^	52 (11%)^∗$^	11 (2%)	132 (29%)^∗$^	116 (25%)	16 (4%)^∗$^	62 (14%)^∗$^
Men	19 (4%)	16 (4%)	3 (<1%)	70 (15%)	68 (15%)	2 (<1%)	19 (4%)

Total	82 (18%)	68 (15%)	14 (3%)	202 (44%)	184 (40%)	18 (4%)	81 (18%)

*P < 0.05 comparison between women and men; ^$^ *P* < 0.05 comparison between the percentage of women with the percentage of men in RDC/TMD groups; (%) percentage off all respondents.

**Table 5:** Comparison of answers to selected questions of the questionnaire in the group of students exhibiting muscle disorders.

Selected question	Selected RDC/TMD diagnoses		
	GROUP I		
	Muscle disorders		
	I (total)	Ia	Ib
Emotional burden			
Yes	58 (13%)^∗$^	48 (11%)^#$^	10 (3%)
No	24 (5%)	20 (4%)	4 (<1%)
Fatigue and tightness of the facial muscles			
Yes	44 (10%)^∗$^	38 (8%)^#$^	6 (1%)
No	38 (8%)	30 (7%)	8 (2%)
Cervical muscle pain			
Yes	55 (12%)^∗$^	43 (9%)^#$^	12 (3%)^∧$^
No	27 (6%)	25 (5%)	2 (<1%)
Headache			
Yes	50 (11%)^∗$^	39 (9%)^#$^	11 (2%)^∧$^
No	32 (7%)	29 (6%)	3 (<1%)

*P < 0.05 comparison between group I (total) and students without muscle disorders; ^#^ *P* < 0.05 comparison between group IIa and students without muscle disorders; ^∧^ *P* < 0.05 comparison between group Ib and students without muscle disorders; ^$^ *P* < 0.05 comparison between percentage of “yes” answers with percantage of “no” answers in the RDC/TMD group; (%) percentage off all respondents.
